# Gestational exposure to environmental chemicals and epigenetic alterations in the placenta and cord blood mononuclear cells

**DOI:** 10.1186/s43682-024-00027-7

**Published:** 2024-06-30

**Authors:** Jagadeesh Puvvula, Joseph M. Braun, Emily A. DeFranco, Shuk-Mei Ho, Yuet-Kin Leung, Shouxiong Huang, Xiang Zhang, Ann M. Vuong, Stephani S. Kim, Zana Percy, Antonia M. Calafat, Julianne C. Botelho, Aimin Chen

**Affiliations:** 1 Department of Biostatistics, Epidemiology and Informatics, Perelman School of Medicine, University of Pennsylvania, Philadelphia, PA USA; 2 Department of Epidemiology, Brown University, Providence, RI USA; 3 Department of Obstetrics and Gynecology, College of Medicine, University of Kentucky, Lexington, KY USA; 4 Department of Pharmacology and Toxicology, College of Medicine, University of Arkansas for Medical Sciences, Little Rock, AR USA; 5 Pathogen-Host Interaction Program, Texas Biomedical Research Institute, San Antonio, TX USA; 6 Department of Environmental & Public Health Sciences, College of Medicine, University of Cincinnati, Cincinnati, OH USA; 7 Department of Epidemiology and Biostatistics, School of Public Health, University of Nevada Las Vegas, Las Vegas, NV USA; 8 Health Research, Battelle Memorial Institute, Columbus, OH USA; 9 National Center for Environmental Health, U.S. Centers for Disease Control and Prevention, Atlanta, GA USA

**Keywords:** Polycyclic aromatic hydrocarbons, Phenol, Phthalate, Placenta, Cord blood, Methylation

## Abstract

**Background:**

Exposure to environmental chemicals such as phthalates, phenols, and polycyclic aromatic hydrocarbons (PAHs) during pregnancy can increase the risk of adverse newborn outcomes. We explored the associations between maternal exposure to select environmental chemicals and DNA methylation in cord blood mononuclear cells (CBMC) and placental tissue (maternal and fetal sides) to identify potential mechanisms underlying these associations.

**Method:**

This study included 75 pregnant individuals who planned to give birth at the University of Cincinnati Hospital between 2014 and 2017. Maternal urine samples during the delivery visit were collected and analyzed for 37 biomarkers of phenols (12), phthalates (13), phthalate replacements (4), and PAHs (8). Cord blood and placenta tissue (maternal and fetal sides) were also collected to measure the DNA methylation intensities using the Infinium HumanMethylation450K BeadChip. We used linear regression, adjusting for potential confounders, to assess CpG-specific methylation changes in CBMC (*n* = 54) and placenta [fetal (*n* = 67) and maternal (*n* = 68) sides] associated with gestational chemical exposures (29 of 37 biomarkers measured in this study). To account for multiple testing, we used a false discovery rate q-values < 0.05 and presented results by limiting results with a genomic inflation factor of 1±0.5. Additionally, gene set enrichment analysis was conducted using the Kyoto Encyclopedia of Genes and Genomics pathways.

**Results:**

Among the 29 chemical biomarkers assessed for differential methylation, maternal concentrations of PAH metabolites (1-hydroxynaphthalene, 2-hydroxyfluorene, 4-hydroxyphenanthrene, 1-hydroxypyrene), monocarboxyisononyl phthalate, mono-3-carboxypropyl phthalate, and bisphenol A were associated with altered methylation in placenta (maternal or fetal side). Among exposure biomarkers associated with epigenetic changes, 1-hydroxynaphthalene, and mono-3-carboxypropyl phthalate were consistently associated with differential CpG methylation in the placenta. Gene enrichment analysis indicated that maternal 1-hydroxynaphthalene was associated with lipid metabolism and cellular processes of the placenta. Additionally, mono-3-carboxypropyl phthalate was associated with organismal systems and genetic information processing of the placenta.

**Conclusion:**

Among the 29 chemical biomarkers assessed during delivery, 1-hydroxynaphthalene and mono-3-carboxypropyl phthalate were associated with DNA methylation in the placenta.

**Supplementary Information:**

The online version contains supplementary material available at 10.1186/s43682-024-00027-7.

## Background

Pregnant individuals are exposed to a spectrum of environmental chemicals that may increase their risk of adverse maternal or fetal health outcomes [1]. Among these chemicals, metabolites of polycyclic aromatic hydrocarbons (PAHs), phenols, and phthalates are frequently detected in the U.S. pregnant population [1]. Despite the non-persistent nature of these chemicals, exposures during pregnancy have been associated with adverse birth outcomes [2–8]. Exposure to exogenous endocrine-disrupting chemicals (EDC) such as phenols, phthalates, and PAHs (present in the environment, food, and consumer products) may result in homeostatic, reproductive, and developmental dysregulation [9–11].

Environmental chemical exposures during pregnancy have the potential to influence up to three generations that encompass the mother, developing child, and developing gametes within the developing embryo/fetus [12–14]. Exposure to EDCs during fetal development may result in adverse health outcomes during adulthood, and these environmental insults can be observed from the alterations in DNA methylation [12]. PAHs, phthalates, and phenols, exhibit endocrine-disrupting properties by mimicking or altering the synthesis/metabolism/transport/action/excretion of hormones (thyroid, insulin, estrogen, androgens), which in turn could disrupt hormonal physiology related to epigenetic pathways [9, 15, 16]. Several studies evaluated the associations between gestational exposure to phthalates, phenols, and cord blood global methylation, suggesting inconsistent patterns in CpG-specific associations [17–22].

However, a limited number of studies have examined placenta methylation changes in relation to gestational exposure to environmental chemicals [23]. A few of these studies highlighted associations between gestational phenol/phthalate biomarkers and alterations in the global DNA methylations(DNAm), which are limited to the fetal side of the placenta [22]. Given the importance of the placenta, which plays a critical role in nutrient regulation, gas/waste exchange, and hormone secretion, and the relevance of placental epigenetic alterations to newborn phenotyping, this study focuses on cord blood mononuclear cells (CBMC) and the placenta methylation [23–26]. In the current study, we quantified the global DNAm of CBMC and maternal/fetal side of placenta changes associated with non-persistent EDC biomarkers to highlight potential developmental origins of diseases.

## Materials and methods

### Study participants

We enrolled 75 pregnant individuals between August 2014 and September 2017 from a labor and delivery unit at the University of Cincinnati Medical Center, Cincinnati, Ohio. The Institutional Review Board at the University of Cincinnati reviewed and approved the research protocol. The study participants were women with singleton pregnancies, aged between 18 and 45 years, who were admitted for childbirth and provided written consent for their involvement in the study at enrollment. Individuals with a medical history involving diabetes, thyroid disorders, cardiovascular, renal, hepatic conditions, cancers affecting pregnancy, or severe maternal or fetal complications at the time of recruitment were excluded from participation.

We collected 30 mL of maternal urine during the hospital visit for childbirth. Additionally, 10 mL of cord blood and placenta tissue from the maternal and fetal sides were collected. Detailed information on extracting the maternal and fetal side of the placenta tissue is available in the supplement information (SM1). The biospecimens were stored at -80 °C and shipped to labs on dry ice overnight; biospecimens were kept frozen until analysis. Demographic, medical, tobacco use during pregnancy, pre-pregnancy body mass index (BMI), and pregnancy outcome data for both the mother and infant were obtained through questionnaires and a review of medical records.

### Chemical exposure assessment

The urinary concentrations of eight PAH metabolites (1-hydroxynaphthalene, 2-hydroxynaphthalene, 2-hydroxyfluorene, 1-hydroxyphenanthrene, 2,3-hydroxyphenanthrene, 4-hydroxyphenanthrene, 9-hydroxyphenanthrene, and 1-hydroxypyrene) were quantified at NSF International – Applied Research Center in Ann Arbor, MI using ultra-high-performance liquid chromatography tandem mass spectrometry [27]. The assays were implemented using a Thermo Scientific Transcend TXII Turbulent Flow system coupled with a Thermo Scientific Vantage triple quadrupole mass spectrometer using multiple reaction monitoring in negative mode.

We also quantified urinary concentrations of 29 biomarkers of triclocarban, phenols, parabens, phthalates, di(2-ethylhexyl) terephthalate, and di(isononyl) cyclohexane-1,2-dicarboxylate using on-line solid-phase extraction coupled with high-performance liquid chromatography isotope dilution tandem mass spectrometry [28, 29]. Concentrations of these biomarkers were quantified at the Centers for Disease Control and Prevention (CDC), Atlanta, GA. The involvement of the CDC laboratory did not constitute engagement in human-participant research.

The limit of detection (LOD) for each chemical biomarker is listed in Table 1. Biomarker concentrations below LOD were imputed with LOD divided by the square root of two [30]. Among the 37 chemical biomarkers quantified from the maternal urine, we excluded eight biomarkers. Specifically, we excluded four phthalate/phthalate replacement biomarkers (mono-2-ethyl-5-carboxypentyl phthalate, mono-2-ethyl-5-hydroxyhexyl phthalate, mono-2-ethyl-5-oxohexyl phthalate, and mono-2-ethylhexyl phthalate) and three parabens (ethyl paraben, methyl paraben, propyl paraben) detected in all study participants due to their potential exposure from medical products during labor and delivery. Additionally, we excluded mono-isononyl phthalate, which was detected in 6% of participants, as outlined in Table 1.

**Table 1:** Summary statistics of maternal urinary biomarkers (*n* = 72)

Chemical biomarker	LOD	% < LOD	Median (IQR)**
1-Hydroxynaphthalene*	0.05	4.0	142.1 (74.5-303.3)
2-Hydroxynaphthalene*	0.05	0.0	4380.7 (2490.5-8461.6)
2-Hydroxyfluorene*	0.01	1.3	123.9 (73.4-232.7)
1-Hydroxyphenanthrene*	0.01	0.0	121.9 (74.7-194.4)
2,3-Hydroxyphenanthrene*	0.02	0.0	101.2 (68.9-154.4)
4-Hydroxyphenanthrene*	0.01	12.0	14.3 (4.2–20.4)
9-Hydroxyphenanthrene*	0.01	8.0	17.7 (10.4–33.3)
1-Hydroxypyrene*	0.01	1.3	120.3 (70.9–173.0)
Monoethyl phthalate	1.2	0	13.1 (4.9–47.2)
Mono-n-butyl phthalate	0.4	5.3	3.8 (1.9–12.8)
Mono-isobutyl phthalate	0.8	21.3	3.0 (0.9–11.5)
Monobenzyl phthalate	0.3	8.0	1.7 (0.9–7.6)
Mono-3-carboxypropyl phthalate	0.4	41.3	0.6 (< LOD-1.8)
Mono-2-ethyl-5-carboxypentyl phthalate^b^	0.4	0	112 (43.3-269.5)
Mono-2-ethyl-5-hydroxyhexyl phthalate^b^	0.4	0	69.7 (26.6–198.0)
Mono-2-ethyl-5-oxohexyl phthalate^b^	0.2	0	61.0 (16.35–160.0)
Mono-2-ethylhexyl phthalate^b^	0.8	4.0	45.3 (10.9-112.5)
Mono-isononyl phthalate^a^	0.9	93.3	< LOD
Monooxononyl phthalate	0.4	21.3	1 (0.5–2.9)
Mono carboxyisooctyl phthalate	0.3	10.7	1 (0.3–3.3)
Mono carboxyisononyl phthalate	0.2	0	3.3 (1.5–10.5)
Mono-2-ethyl-5-carboxypentyl terephthalate	0.2	0	20.1 (5.9–59.3)
Mono-2-ethyl-5-hydroxyhexyl terephthalate	0.4	33.3	1.4 (< LOD-6.3)
Cyclohexane-1,2-dicarboxylic acid, monocarboxy isooctyl ester	0.5	80	< LOD
Cyclohexane-1,2-dicarboxylic acid, monohydroxy isononyl ester	0.4	64	< LOD (< LOD-0.7)
2,4-Dichlorophenol	0.1	9.3	0.5 (0.2–1.1)
2,5-Dichlorophenol	0.1	6.7	1.2 (0.5–6.8)
Benzophenone-3	0.4	4.0	6.7 (2.4–25.7)
Bisphenol A	0.2	4.0	1.0 (0.5-2.0)
Bisphenol F	0.2	56	< LOD (< LOD-0.5)
Bisphenol S	0.1	24	0.2 (0.1–0.7)
Methyl paraben^b^	1.0	0	376.7 (68.5-1214.6)
Ethyl paraben^b^	1.0	26.7	2.1 (< LOD-16.2)
Propyl paraben^b^	0.1	1.3	21.1 (3.9–84.1)
Butyl paraben	0.1	72	< LOD
Triclocarban	0.1	52	< LOD (< LOD-0.6)
Triclosan	1.7	52	< LOD(< LOD-4.2)

^a^Excluded in further analysis due to relatively low detection frequencies

^b^Excluded due to relatively high concentrations that are likely due to use of medical tubing/medications/wipes at delivery

*Unit of concentration for PAH biomarkers is nanogram/gram creatinine (ng/g creatinine); concentration unit for all other biomarkers is microgram/gram creatinine (µg/g creatinine)

**Biomarker concentrations standardized with urine creatinine concentration

### DNA methylation, preprocessing, and cell deconvolution

We extracted DNA using a DNeasy Blood and Tissue kit with RNase A, following the manufacturer’s protocols. The DNA was then prepared for the array following the Illumina Infinium HD Assay Methylation Protocol Guide. We randomly assigned DNA samples to well plates within a chip, as visualized in Figure S1, to reduce potential batch effects. Bisulfite conversion of the DNA was performed using the EZ DNA Methylation Kit (Zymo Research, Irvine, CA), and DNA methylation levels were measured using Infinium HumanMethylation450K BeadChip (Illumina, San Diego, CA) by the Genomics, Epigenomics and Sequencing Core at the University of Cincinnati.

Raw methylation data were processed following the steps recommended by Wilhelm-Benartzi et al. using the minfi Bioconductor R-package [31, 32]. Quality control steps include removing samples with > 5% high detection p-values (p-value > 1e-7; *n* = 0) and probes that failed in one or more samples using the same p-value cutoff [33]. Background correction with dye-bias normalization was performed using the normal-exponential out-of-band (noob) procedure [34]. Additionally, bias resulting from type-2 probe values was normalized using the beta-mixture quantile normalization method [35] and followed by potential batch effect correction using an empirical Bayes framework available through the ComBat function [36]. Additionally, the CpG probes located on sex chromosomes, enriched at single-nucleotide polymorphisms (SNPs), and those prone to cross-reactivity were excluded [37]. Following these preprocessing steps, we included 418,997 CpGs from CBMC, 415,604 from the fetal side of the placenta, and 412,460 from the maternal side of the placenta for further downstream analysis. We estimated the proportions of five cell types [T-lymphocytes (CD8T, CD4T), NK cells (CD56+), B-cells (CD19+), and monocytes (CD14+)] using CBMC methylation intensities using Houseman’s reference-based (cord blood – HM450) method [38, 39]. Additionally, we estimated the proportions of six cell types [trophoblasts, syncytiotrophoblast, stromal, Hofbauer, endothelial, and nucleated red blood cells (nRBC)] based on placenta methylation intensities using a reference-based (third-trimester placenta) approach [40, 41].

These CpG sites were annotated to genes using the University of California, Santa Cruz Genome Browser (Human GRCh37/hg19) database [42, 43]. In subsequent analysis, we used M-values (log2 ratio of the intensities of methylated versus unmethylated probe) to enhance statistical validity [44].

### Statistical analysis

#### Differential methylated CpG positions

We identified differentially methylated CpGs using the 22 chemical biomarkers (urine creatinine adjusted and log10-transformed) measured in maternal urine as independent variables and the M-values (methylation intensity) of CpGs as dependent variables. Additionally, the associations between 7 maternal chemical biomarkers and the methylation intensities were assessed, considering chemical biomarkers as binary variables (detected or not-detected) due to a relatively lower detection frequency (detected among 20–40% of participants) among the study participants. These associations were adjusted for covariates based on prior literature, including maternal age, race, education, tobacco use during pregnancy, BMI, child sex, and cell type proportions [45, 46]. Associations between 22 biomarker concentrations measured on a continuous scale and M-value per CpG site were assessed using multiple linear regression [47]. Additionally, the association between seven biomarkers considered as binary variables and M-values per CpG site were tested using the ANOVA test [47]. Among the 75 subjects in this study, we limited our sample size to 54 while analyzing CBMC samples, 67 fetal side placenta, and 68 maternal side placenta, due to the availability of DNA methylation (DNAm) and chemical biomarker data. To handle the false positives as a result of multiple tests, we considered CpG sites with Benjamini-Hochberg’s false discovery rate (FDR) at the 5% level (q-values < 0.05) as statistically significant [48]. To expand on biological phenotype interpretations, we performed downstream analysis using CpGs with FDR q-value < 0.05 and for chemical biomarkers with genomic inflation factor (λ) 1 ± 0.5, to minimize false positives [49]. The inflation factor is the ratio between the median of the observed and expected chi-squared statistic [50]. Although the λ value close to one reflects no evidence of inflation (false positives), we considered a liberal threshold of 1 ± 0.5, due to the exploratory study objective [51].

#### Gene enrichment, pathways, and phenotype inference

Gene enrichment analysis was performed assuming Wallenius noncentral hypergeometric distribution, using the missMethyl R-package considering the Kyoto Encyclopedia of Genes and Genomes (KEGG) pathways [50]. Additionally, we performed a sensitivity analysis using the 500 CpGs with relatively lowest q-values for gene enrichment and highlighted the pathways with q-values < 0.1.

The analyses and preprocessing throughout this article were performed using R-packages: minfi 1.44, sva 3.46, wateRmelon 2.4, planet 1.8, CpGassoc 2.6, missMethyl 1.32, R 4.2 [52].

#### Associations between maternal biomarkers and estimated cell composition

We additionally explored potential associations between maternal chemical biomarkers and cell compositions estimated using DNAm intensities from CBMC/placenta. We tested for normality distribution assumption for estimated cell proportions using the Shapiro-Wilk test [53]. These associations were assessed using a multiple linear regression approach adjusting for maternal age, race, education, tobacco use during pregnancy, BMI, and child sex. The beta coefficients with a corresponding 95% confidence interval without a null value were considered statistically significant. Additionally, we performed a sensitivity analysis by transforming both the maternal urinary chemical biomarker concentrations and cell proportions to log_10_ scale to address potential non-linear trends of the outcome variables.

## Results

This study involved pregnant individuals with a median age of 29 years (interquartile range (IQR): 25–32) at the time they gave birth, and the majority (83.3%) had previous pregnancies (Table S1). About half of the subjects were non-Hispanic Black females (47.2%). The median BMI of the study participants was 25.9 kg/m^2^ (IQR: 22.5–30.5), and 17% self-reported smoking tobacco during their pregnancy. Most study participants (75%) attained less than a 4-year college education, and 64% had a household income below the median for Cincinnati, OH. All the participants included in this study had full-term pregnancies, with gestational ages ranging from 37 to 41 weeks. We observed correlations between maternal urinary biomarkers that varied between − 0.29 and 0.78 (Figure S2). Overall, the biomarkers within a chemical class showed relatively strong positive correlations.

### Differential methylation of CpG sites

Among the epigenetic-wide associations (EWAS) assessed with 22 chemical biomarkers, we observed statistically significant associations with differential methylation of CpG sites for 7 biomarkers (Table S2). These biomarkers encompass PAHs (1-hydroxynaphthalene, 2-hydroxynaphthalene, 2-hydroxyfluorene, 2,3-hydroxyphenanthrene, 4-hydroxyphenanthrene, 1-hydroxypyrene); phenols (bisphenol A); and phthalates (mono-3-carboxypropyl phthalate [MCPP]), mono-2-ethyl-5-hydroxyhexyl terephthalate, mono-2-ethyl-5-carboxypentyl terephthalate, mono carboxy-isononyl phthalate). Notably, 2 phthalate metabolites (mono-2-ethyl-5-carboxypentyl terephthalate, and mono-2-ethyl-5-hydroxyhexyl terephthalate) associated with maternal side CpG methylation; 2,3-hydroxyphenanthrene association with fetal side placenta methylation; and 2-hydroxynaphthalene, MCPP associations with CBMC methylation were not highlighted in this study due to higher lambda values (genomic inflation factor).

**Fig. 1:**
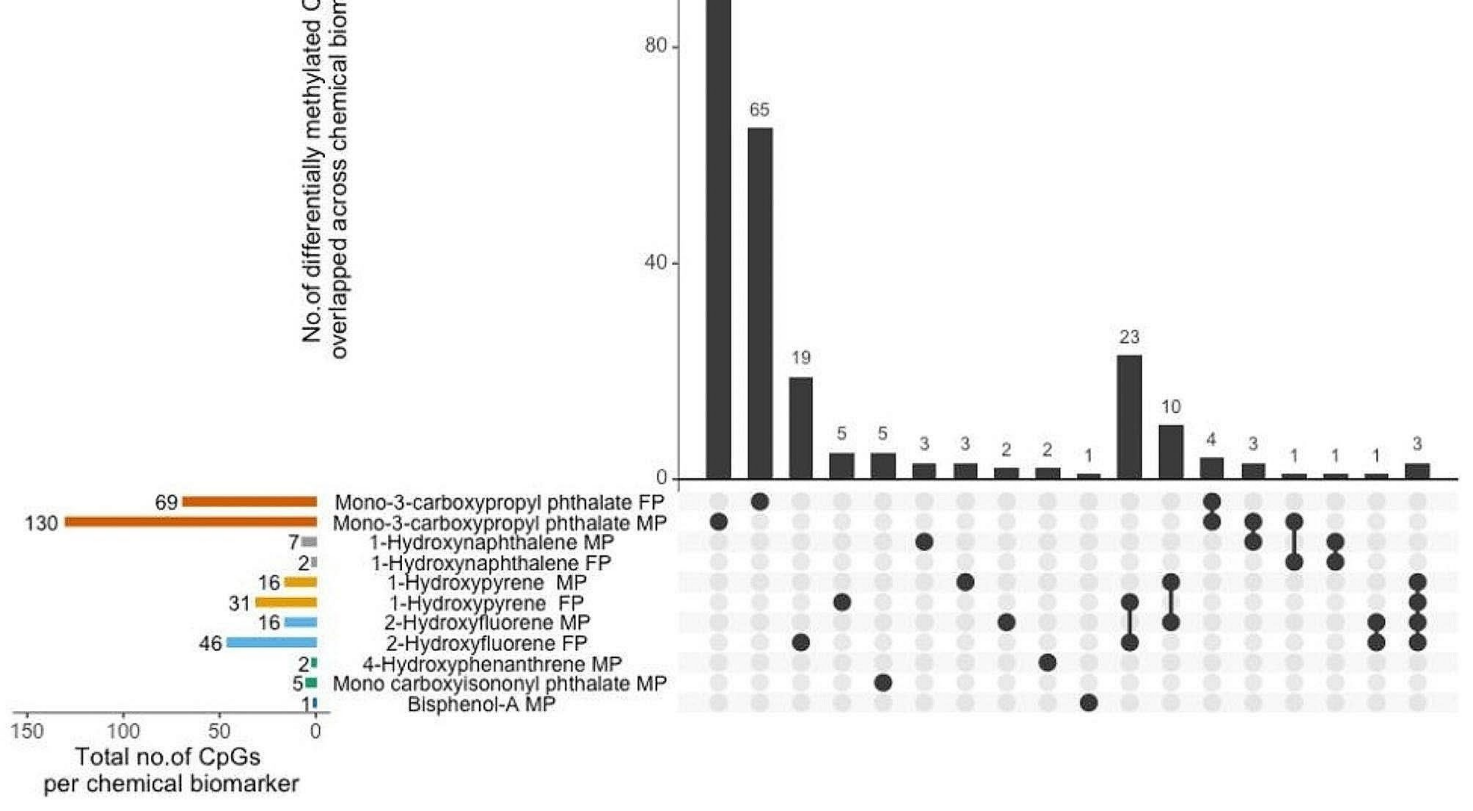
Summary of CpG-specific associations (q-value < 0.05) with gestational chemical biomarkers. Horizontal bars represent the total number of CpGs associated with chemical biomarkers by methylation sample source. MP-maternal side placenta, FP-fetal side placenta. Vertical bars represent the number of CpG associations that overlap across and are exclusive to a chemical biomarker

**Fig. 2:**
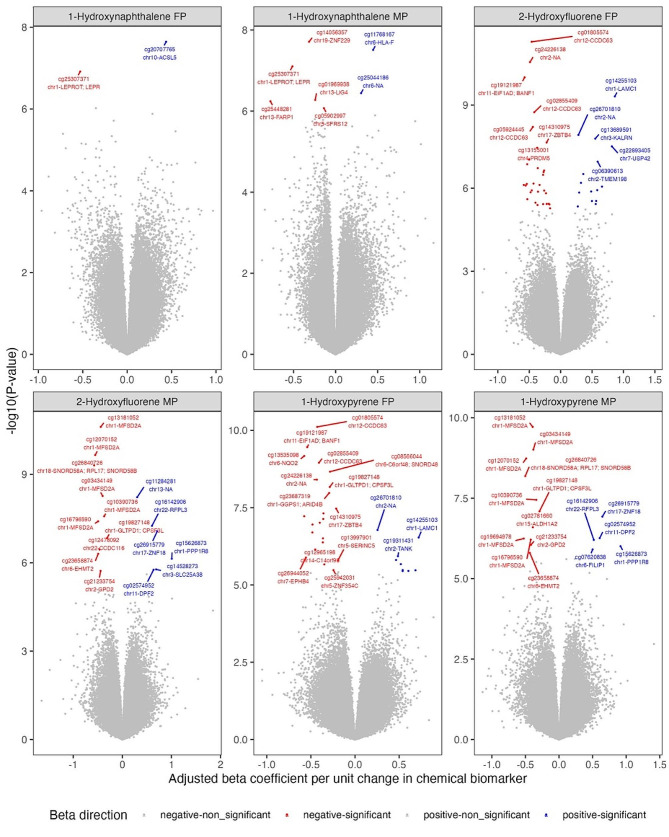
Association between PAH biomarkers concentrations and CpG methylation intensities. This figure covers 1-hydroxynaphthalene and 2-hydroxyfluorene. Methylation intensities from fetal-side placenta (FP) and maternal-side placenta (MP). The x-axis represents the beta coefficient values, and the y-axis represents raw p-values on the -log10 scale. The CpG ID, chromosome position, and UCSC Reference Gene names were labeled. CpG-specific associations with q-values < 0.05 were color-coded in red (hypomethylation) and blue (hypermethylation)

**Fig. 3:**
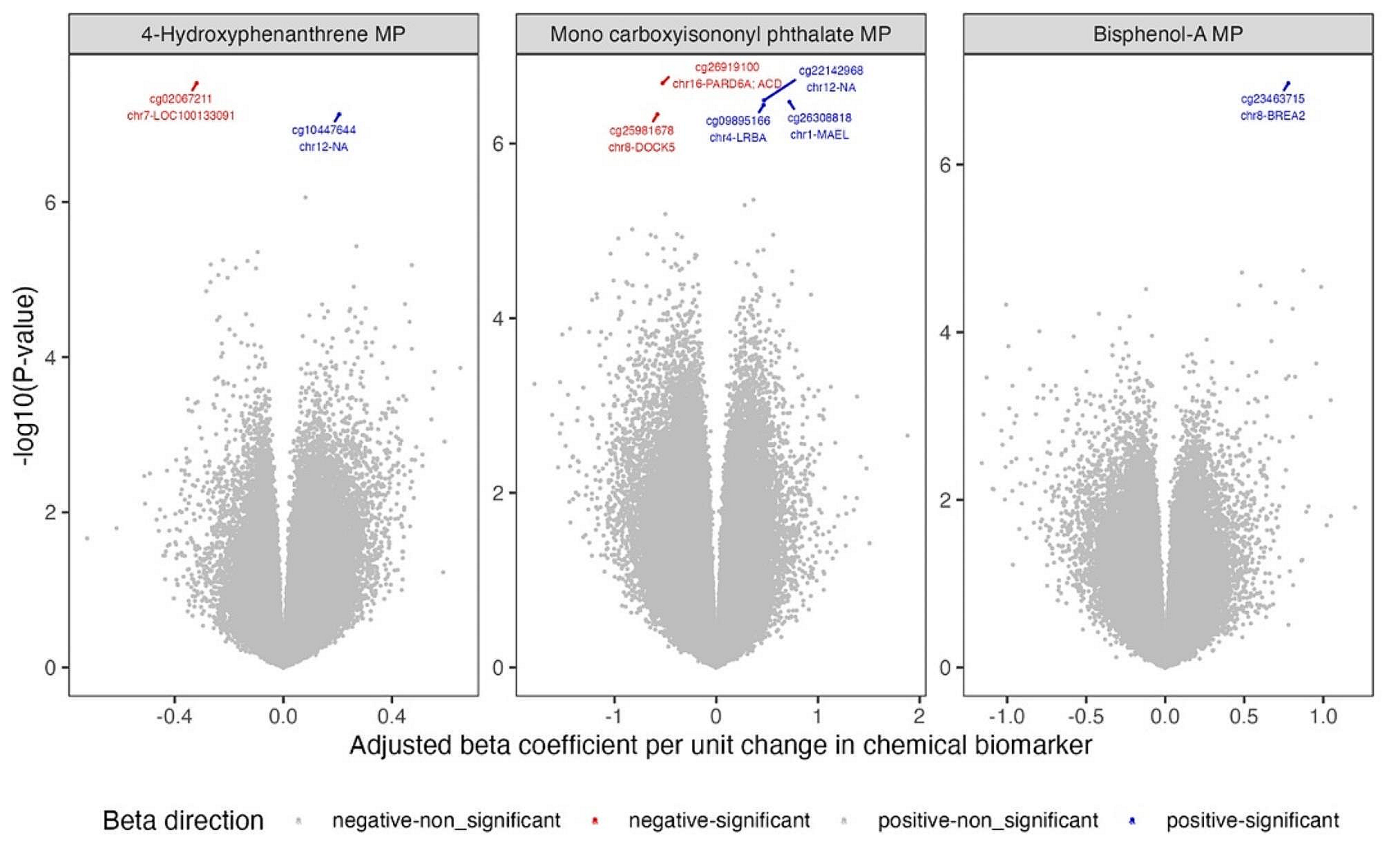
Association between select chemical biomarkers concentrations and CpG methylation intensities. MP – Methylation intensities from the maternal side of the placenta tissue

Among the 22 biomarkers assessed using the linear regression approach, we observed a relatively higher number (*n* = 46) of CpGs from the fetal side of the placenta associated with 2-hydroxyfluorene concentrations (Fig. 1). Among these associations, most (11 of 46) were related to hypomethylation of CpG sites that are within 200 base pairs of the promotor [TSS (transcription start site)] region of genes—followed by 1-hydroxypyrene associated with 31 CpGs from the fetal side of the placenta. Nine of the 31 CpG sites associated with 1-hydroxypyrene were hypomethylated and within 200 base pairs of the TSS region of a gene. There were 23 CpG sites from the fetal side of the placenta that were commonly associated with 1-hydroxypyrene and 2-hydroxyfluorene (Figs. 2 and 3). Similarly, a higher number of CpG sites (*n* = 10) from the maternal side of the placenta were commonly associated with 1-hydroxypyrene and 2-hydroxyfluorene.

Gestational urinary concentrations of 2-hydroxyfluorene were associated with hypomethylation of seven CpG sites from the fetal side of the placenta, which are located at the promotor region of genes zinc finger protein 345 C (*ZNF354C*) and coiled-coil domain containing 63 (*CCDC63*). The CpG sites closest to the *ZNF354C* gene were in the TSS1500 region and were labeled as cg19761115, cg10864596, and cg12623648. For the *CCDC63* gene, the closest CpG sites were in the TSS200 region and were labeled as cg05924445, cg10995082, cg01805574, and cg02855409.

Furthermore, we observed associations between 2-hydroxyfluorene and hypomethylation of 4 of 16 CpG sites (cg03434149, cg16796590, cg13181052, cg10390736) from the maternal side of the placenta that are within 200 base pairs of the TSS region of major facilitator superfamily domain containing 2 A (*MFSD2A*) gene. Additionally, there were associations between 1-hydroxypyrene and hypomethylation of 5 of 16 CpG sites (cg19694978, cg03434149, cg16796590, cg13181052, cg10390736) located within 200 base pairs of the TSS region of *MFSD2A* gene.

Highlighting seven biomarkers assessed using the ANOVA test, we observed that MCPP was associated with differential methylation of the placenta. About 130 CpGs from the maternal side of the placenta and 69 CpGs from the fetal site of the placenta were associated with maternal MCPP concentrations. Among these CpGs associated with MCPP, 34 from the maternal side of the placenta and 15 from the fetal side of the placenta were located in the TSS regions. Here, we highlighted the results with more than a CpG located in the TSS region and associated with MCPP concentrations. Among the CpGs associated with MCPP on the maternal side of the placenta, four specific CpGs (cg05180121, cg17114573, cg08216792, cg22492943) were identified within 200 base pairs of the TSS region of the DNA Ligase 4 (*LIG4*) gene. On the fetal side of the placenta, two CpGs (cg08231348, cg11792516) associated with MCPP were located within 200 base pairs of the TSS region of the hook microtubule tethering protein (*HOOK1*) gene, and two CpGs (cg02788400, cg05505803) located within 200 base pairs of the TSS region of the DAZ (deleted in azoospermia) interacting zinc finger protein (*DZIP1*) gene.

### Enrichment of Kyoto Encyclopedia of Genes and Genomes (KEGG) pathways

None of the KEGG pathways enriched with FDR q-values below 0.1. Therefore, we highlighted the pathways based on raw p-values less than 0.01. Our findings from the enrichment analysis suggest that 1-hydroxynaphthalene urinary concentrations were associated with 6 KEGG pathways using CpG methylation intensities from the maternal and fetal side of the placenta (Table 2). Of these, there were four pathways enriched for CpGs from the fetal side of the placenta that represent lipid metabolism (fatty acid biosynthesis, degradation, and metabolism) and cellular process (ferroptosis). At the same time, two pathways were enriched while using CpGs from the maternal side of the placenta that represent viral infectious disease (Herpes simplex virus-1) and genetic information replication or repair (non-homologous end-joining). In contrast, results from our sensitivity analysis using the top 500 affected CpG sites suggest associations between CpGs from the maternal side placenta and 1-hydroxynaphthalene were through fatty acid metabolism pathway (Table S3).

**Table 2:** KEGG pathways enriched at p-value < 0.01 using CpGs with q < 0.05

Chemical biomarker	Sample	Pathway ID	Description	*p*-value
1-Hydroxynaphthalene	FP	hsa00061	Fatty acid biosynthesis	2.86e-03
1-Hydroxynaphthalene	FP	hsa00071	Fatty acid degradation	6.25e-03
1-Hydroxynaphthalene	FP	hsa04216	Ferroptosis	6.72e-03
1-Hydroxynaphthalene	FP	hsa01212	Fatty acid metabolism	9.38e-03
1-Hydroxynaphthalene	MP	hsa03450	Non-homologous end-joining	4.96e-03
1-Hydroxynaphthalene	MP	hsa05168	Herpes simplex virus-1 infection	7.93e-03
Mono-3-carboxypropyl phthalate	MP	hsa03450	Non-homologous end-joining	2.2e-03
Mono-3-carboxypropyl phthalate	MP	hsa04613	Neutrophil extracellular trap formation	9e-03

hsa - homo sapiens; FP: fetal-side placenta; MP: maternal-side placenta

There were seven CpG sites (cg25448281, cg11768167, cg25307371, cg01969938, cg25044186, cg05902997, cg14056357) from the maternal side of the placenta that were associated with 1-hydroxynaphthalene. Most of these CpGs (6 of 7) associated with 1-hydroxynaphthalene were hypomethylated. These hypomethylated CpGs are located closer to the promotor region of *LEPROPT/LEPR* (cg25307371), *LIG4* (cg01969938), *SFRS12* (cg05902997), and *ZNF229* (cg14056357) genes, except for cg25448281, which is located at the end of a protein-coding sequence (3’UTR) of *FARP1* gene.

There were relatively lower (*n* = 2; cg20707765, cg25307371) CpG site-specific associations from the fetal side of the placenta that correspond to leptin receptor overlapping transcript (*LEPROT/LEPR*) and acyl-CoA synthetase long-chain family member 5 (*ACSL5*), genes. Urinary concentrations of 1-hydroxynaphthalene were associated with hypermethylation of cg20707765, located within 1500 base pairs of the TSS region of gene *ACSL5*. At the same time, 1-hydroxynaphthalene was associated with hypomethylation of cg25307371, located within 1500 base pairs of gene *LEPROT/LEPR*.

Furthermore, CpGs from the maternal side of the placenta, associated with MCPP concentrations, were associated with genetic information processing (non-homologous end-joining [NHEJ]) and organismal systems (neutrophil extracellular trap formation). The NHEJ could be attributable to the four CpGs associated with MCPP from the maternal side of the placenta, positioned proximally to the TSS of the *LIG4* gene. Additionally, enrichment of the neutrophil extracellular trap formation could be related to the CpG (cg23207054) from the maternal side of the placenta that is located within 200 base pairs of the colony-stimulating factor 3 (*CSF3)* gene and associated with the MCPP concentrations.

### Associations between maternal chemical biomarkers and estimated cell composition

Most of these cell proportions estimated from the DNA methylation intensities showed normal distributions, except for NK-cells from CBMC, Hofbauer, and nRBC cells from the fetal side of the placenta that are slightly skewed (Figure S3). We observed a weak association pattern between chemical biomarkers and estimated cell compositions in CBMC/placenta (Figure S4-A). Among the cells estimated using DNAm intensities in CBMC, we observed statistically significant negative associations between biomarkers of PAHs and B-cells. Similarly, we observed statistically significant negative associations between biomarkers of PAHs and nRBC cells estimated from the DNAm intensities measured from the maternal side of the placenta. However, we also observed statistically significant positive associations between phthalate biomarkers and CD4T cells from CBMC as well as Trophoblasts from the maternal side of the placenta. Moreover, transforming the cell proportions on a log_10_ scale, we identified additional negative associations between 2-hydroxyfluorene, 2,3-hydroxyphenanthrene, monobenzyl phthalate, and B-cell proportions estimated from CBMC; 1-hydroxyphenanthrene and nRBCs from maternal placenta; monobenzyl phthalate and stromal cell proportions estimated from maternal placenta (Figure S4-B). The results presented here for NK cells from cord blood, nRBC from the fetal placenta, and Hofbauer cells from the maternal/fetal placenta require caution in interpretation since the cell proportions were not normally distributed (Table S4).

## Discussion and conclusions

Among the 29 environmental chemical biomarkers assessed for EWAS, 11 biomarkers (6 PAHs, 4 phthalates, and one phenol) were associated with differential CpG methylation levels. Across the sample media (CBMC, fetal side placenta, and maternal side placenta), the differential methylation associations were prominent with the fetal side of the placenta. Our gene enrichment analysis results indicate associations between 1-hydroxynaphthalene, MCPP, and placental metabolism pathways. Our results highlight plausible mechanisms linking exposure to environmental chemicals during pregnancy and placenta development.

Focusing on the 1-hydroxynaphthalene and CpG methylation while using the fetal side of the placenta, two CpG sites are closest to *LEPROT/LEPR* (cg25307371), and *ACSL5* (cg20707765) genes that were differentially methylated. The *LEPROT/LEPR* gene is associated with obesity and *ACSL5* – obesity and gastrointestinal conditions [54, 55]. There are studies focused on the associations between PAH metabolites and DNA methylation, suggesting altered global methylation patterns, epigenetic aging, chronic diseases (pulmonary, cardiovascular), endocrine disruption, nervous system disorders, and cancer [56–60]. Additionally, gestational measurements of the PAH metabolites were associated with methylation of *ACSL3* from cord white blood cells, which was found to be a potential biomarker for environmentally-related asthma [61].

Summarizing our findings from the gene enrichment analysis, we observed associations between differentially methylated CpGs from the fetal side of the placenta related to 1-hydroxynaphthalene and lipid metabolism and cellular process. Alterations in lipid metabolism pathways may result in adverse metabolic conditions. Highlighting on the MCPP and CpG methylation from the maternal side of the placenta, there were 34 differentially methylated CpGs in the TSS region representing 31 distinct genes. Furthermore, our findings from the enrichment analysis suggested associations between CpGs from the maternal side of the placenta associated with MCPP and genetic information processing (NHEJ) and organismal systems (neutrophil extracellular trap formation) pathways. The enrichment of these pathways could be relevant to the differential methylation of four CpGs that are located near the TSS region of the *LIG4* gene and a CpG that is located near the TSS region of the *CSF3* gene. Previous studies have implicated that the *LIG4* gene is associated with phenotypic conditions such as metatarsus adductus, abnormality of chromosome stability, abnormality in bone marrow cell morphology, acute lymphoblastic leukemia, and thrombocytopenia [54, 55]. Additionally, the *CSF3* gene is associated with the production and differentiation of granulocytes and macrophages [54, 55].

In contrast to our primary findings, which revealed associations between 1-hydroxynaphthalene and changes in lipid metabolism with the fetal-side placenta, our sensitivity analysis, using 500 CpGs with the lowest p-values, indicated associations between 1-hydroxynaphthalene and altered fatty acid metabolism with the maternal-side placenta. Overall, we observed associations between 1-hydroxynaphthalene, mono-3-carboxypropyl phthalate and differential placenta methylation, which could lead to alterations in fatty acid metabolism, genetic information processing and organismal systems. Similar results have been presented in the literature, suggesting associations between PAH metabolites and metabolic syndrome, cardiovascular conditions, and systemic inflammation [62, 63]. Additionally, a mice-based study suggested prenatal exposure to PAHs and their associated multi-generational (offspring and grand-offspring) epigenetic effects [64]. Furthermore, Jednak et al. identified associations between maternal MCPP exposures and placenta methylation, identifying differential methylation regions of the fibroblast growth factor 12 (*FGF12)* (chr3:192445514–192445539), and the ADP-ribosyltransferase 5 (*ART5)* (chr11:3663491–3663843) genes, suggesting potential alterations in protein function [65]. However, our study’s findings did not align with those reported by Jednak et al., as they measured phthalate biomarkers at ~ 22–29 weeks of pregnancy, contrasting with our study’s measurements at delivery.

Most of the existing literature is supported by the use of whole blood or cord blood to identify global methylation changes associated with organic pollutants, bisphenol A, and polybrominated diphenyl ethers [23]. Our study contributes by exploring potential early-life changes in DNA methylation associated with a spectrum of environmental exposures, using placental tissue. However, we did not find consistent patterns while comparing our results with the existing literature. These inconsistencies could relate to heterogeneity in the sample media to estimate methylation intensities. Although we attempted to minimize false discoveries using the q-value thresholds while identifying differentially methylated CpGs, false positives may still influence our results. Our results are limited to the discovery set analysis and would benefit from a replication study to validate our findings. Additionally, we focused on non-persistent chemical biomarkers measured at a single point in time during the delivery visit, which may not adequately represent exposure during pregnancy and may introduce exposure misclassification bias. The relatively strong correlation pattern between maternal urinary chemical biomarkers may introduce bias due to multi-collinearity.

Furthermore, our findings could be influenced by bias due to unmeasured confounding. Another limitation of our study is the use of the HM450 array that contains substantially fewer type-II probes than compared to the Infinium MethylationEPICv1/v2 chip, which may lead to an underrepresentation of our underlying findings [66, 67]. However, this limitation could be to our advantage to minimize false positives from multiple testing given with our study sample size.

In summary, we observed associations between gestational urinary concentrations of select PAH, phthalate, and phenol biomarkers and differential methylation patterns in the placenta (maternal or fetal). Our findings from the gene enrichment analysis suggest that gestational exposure to some PAH and phthalate metabolites may influence newborn health. However, to confirm these findings, further replication studies are necessary. Additionally, conducting comprehensive multi-omics studies, integrating exposome, epigenetics, transcriptomics, and phenotype data in a larger sample, could enhance our understanding of the biological pathways that underlie the associations between exposure and phenotype.

### Electronic supplementary material

Below is the link to the electronic supplementary material.

Supplementary Material 1

Supplementary Material 2

## Data Availability

The HumanMethylation450K BeadChip data discussed in this publication have been deposited in NCBI’s Gene Expression Omnibus and are accessible through GEO Series accession number GSE269983.
