# Supplementary material for: Gestational exposure to environmental chemicals and epigenetic alterations in the placenta and cord blood mononuclear cells
Source: Epigenetics Commun. 2024 Jun 30;4(1):4. doi: 10.1186/s43682-024-00027-7 (PMC11217138; doi:10.1186/s43682-024-00027-7)
Supplement: Supplementary file 2 — Supplementary Material 2 [file 43682_2024_27_MOESM2_ESM.docx]

Supplemental information: Global metabolomic alterations associated with endocrine-disrupting chemicals among pregnant individuals and newborns

Jagadeesh Puvvula*, Joseph M. Braun, Emily A. DeFranco, Shuk-Mei Ho, Yuet-Kin Leung, Shouxiong Huang, Xiang Zhang, Ann M. Vuong, Stephani S. Kim, Zana Percy, Antonia M. Calafat, Julianne C. Botelho, Aimin Chen

*Corresponding author:

Jagadeesh Puvvula - Department of Biostatistics, Epidemiology and Informatics, Perelman School of Medicine, University of Pennsylvania, Philadelphia, PA; email: [Jagadeesh.Puvvula@pennmedicine.upenn.edu](mailto:Jagadeesh.Puvvula@pennmedicine.upenn.edu)

Contents (12 pages): 4 tables, 4 figures, and methods text

Table S1. Summary of study participant characteristics…………………………….……………2

Table S2. Summary of CpG-specific associations by chemical biomarker…………………..3,4

Table S3. KEGG pathways enriched using 500 CpGs with the lowest q-value and enriched pathways with p-value<0.01…………………………………………………………………………5

Table S4. Normality testing of cell proportion distribution using Shapiro-Wilk test………..…..6

Figure S1. Arrangement of DNA samples to HM450 chips……….………………………….…..7

Figure S2: Spearman correlation coefficients between log_10_ transformed concentrations of maternal urinary biomarkers…………………………………………………………………………8

Figure S3: Distribution of cell proportions estimated using methylation intensities from CBMC and placenta (maternal and fetal sides)……………………………………………………………9

Figure S4-A. Association between maternal biomarker concentrations and estimated cell composition…………………………………………………………………………………………..10

Figure S4-B. Sensitivity analysis – Association between maternal urinary biomarkers and log_10_ transformed cell proportions estimated from methylation intensities……………………11

SM.1. Placenta tissue biopsy for DNA methylation……………………………………………...12

| Table S1. Summary of study participant characteristics (n=72) | |
| --- | --- |
| Variable |  |
| Maternal age at pregnancy^a^ | 29 (25, 32) |
| Maternal BMI^a^ | 25.9 kg/sq.m (22.5, 30.5) |
| Maternal race^b^ | Non-Hispanic Black: 34 (47.2%)  Whites: 29 (40.2%)  Other: 9 (12.6%) |
| Maternal education^b^ | Less than a 4-year college: 54 (75%)  Bachelor’s degree or above: 18 (25%) |
| Household income^b^ | < $20,000: 33 (45.8%)  $20,000-$39,999: 6 (8.3%)  $40,000-$59,999: 7 (9.7%)  ≥ $60,000: 26 (36.1%) |
| Gestational age^a^ | 39.0 weeks (38.5, 39.0) |
| Parity^b^ | 0: 12 (17%)  ≥1: 60 (83%) |
| Self-reported smoking during pregnancy^b^ | Yes: 12 (17%)  No: 60 (83) |
| a-continuous variables presented using median and interquartile range; b-categorical variables presented using count and percentage. Race and household income variables presented here were transformed into binary variables while used as covariates. | |

| Table S2. Summary of CpG-specific associations by chemical biomarker | | | | | | |
| --- | --- | --- | --- | --- | --- | --- |
|  | CBMC (cord blood mononuclear cells) | | Fetal-side placenta | | Maternal-side placenta | |
| Chemical biomarker | λ | # CpG  q<0.05 | λ | # CpG  q<0.05 | λ | # CpG  q<0.05 |
| 1-Hydroxynaphthalene | 0.48 | 2 | 0.83 | 2 | 1.22 | 7 |
| 2-Hydroxynaphthalene | 1.53 | 1 | 1.16 | 0 | 1.13 | 0 |
| 2-Hydroxyfluorene | 1.47 | 0 | 1.39 | 46 | 0.88 | 16 |
| 1-Hydroxyphenanthrene | 0.99 | 0 | 1.52 | 0 | 0.87 | 0 |
| 2,3-Hydroxyphenanthrene | 0.86 | 0 | 1.69 | 1 | 0.88 | 0 |
| 4-Hydroxyphenanthrene | 0.65 | 0 | 1.12 | 0 | 1.01 | 2 |
| 9-Hydroxyphenanthrene | 0.90 | 0 | 1.31 | 0 | 0.93 | 0 |
| 1-Hydroxypyrene | 2.21 | 0 | 1.26 | 31 | 0.84 | 16 |
| Monoethyl phthalate | 1.74 | 0 | 0.81 | 0 | 1.53 | 0 |
| Mono-n-butyl phthalate | 1.06 | 0 | 1.01 | 0 | 1.35 | 0 |
| Mono-isobutyl phthalate | 1.52 | 0 | 0.86 | 0 | 1.41 | 0 |
| Monobenzyl phthalate | 1.81 | 0 | 1.56 | 0 | 1.45 | 0 |
| Monooxononyl phthalate | 1.59 | 0 | 0.83 | 0 | 1.19 | 0 |
| Mono carboxyisooctyl phthalate | 1.91 | 0 | 0.88 | 0 | 0.94 | 0 |
| Mono carboxyisononyl phthalate | 2.27 | 0 | 1.25 | 0 | 1.38 | 5 |
| Mono-2-ethyl-5-carboxypentyl terephthalate | 2.07 | 0 | 1.09 | 0 | 1.78 | 3 |
| Mono-2-ethyl-5-hydroxyhexyl terephthalate | 0.93 | 0 | 0.88 | 0 | 1.67 | 3 |
| 2,4-dichlorophenol | 0.98 | 0 | 1.07 | 0 | 1.18 | 0 |
| 2,5-dichlorophenol | 0.74 | 0 | 0.79 | 0 | 1.16 | 0 |
| Benzophenone-3 | 0.99 | 0 | 0.93 | 0 | 1.04 | 0 |
| Bisphenol A | 0.86 | 0 | 1.43 | 0 | 0.90 | 1 |
| Bisphenol S | 1.35 | 0 | 0.84 | 0 | 0.95 | 0 |
| Bisphenol F^a^ | 1.23 | 0 | 0.88 | 0 | 1.02 | 0 |
| Butyl paraben^a^ | 2.11 | 0 | 1.21 | 0 | 0.92 | 0 |
| Mono-3-carboxypropyl phthalate^a^ | 1.71 | 229 | 0.84 | 69 | 0.86 | 130 |
| Cyclohexane-1,2-dicarboxylic acid, monohydroxy isononyl ester^a^ | 0.77 | 0 | 1.35 | 0 | 1.07 | 0 |
| Cyclohexane-1,2-dicarboxylic acid, monocarboxy isooctyl ester^a^ | 1.35 | 0 | 2.18 | 79 | 1.34 | 0 |
| Triclocarban^a^ | 0.69 | 0 | 1.12 | 0 | 0.94 | 0 |
| Triclosan^a^ | NA | NA | 0.91 | 0 | 1.04 | 0 |
| EWAS sample size: CBMC = 54; fetal-side placenta = 67; maternal-side placenta = 68. Number of CpGs: CBMC=418,997; fetal-side placenta = 415,604; maternal-side placenta = 412,460. λ- genomic inflation factor. a-Urinary biomarkers detected in 20-40% of the study participants and these biomarker concentrations were transformed to binary variables (detected or not-detected) for ANOVA test. | | | | | | |

| Table S3. KEGG pathways enriched using 500 CpGs with the lowest q-value and enriched pathways with p-value<0.01 | | | | | |
| --- | --- | --- | --- | --- | --- |
| Chemical biomarker | Sample | Pathway ID | Description | p-value | q-value |
| 1-hydroxynaphthalene | MP | hsa01040 | Biosynthesis of unsaturated fatty acids | 3.54e-3 | 9.96e-1 |
| 1-hydroxynaphthalene | MP | hsa00061 | Fatty acid biosynthesis | 8.05e-3 | 9.96e-1 |
| 1-hydroxynaphthalene | MP | hsa01212 | Fatty acid metabolism | 1.09e-2 | 9.96e-1 |
| 1-hydroxynaphthalene | MP | hsa00130 | Ubiquinone and other terpenoid-quinone biosynthesis | 1.12e-2 | 9.96e-1 |
| Mono-3-carboxypropyl phthalate | FP | hsa05017 | Spinocerebellar ataxia | 2e-03 | 7.1e-01 |
| MP: Maternal side placenta; FP: Fetal side placenta | | | | | |

| Table S4. Normality testing of cell proportion distribution using Shapiro-Wilk test | | | |
| --- | --- | --- | --- |
| Sample | Cell type | Normal scale | Log_10_ scale |
| Cord blood | CD8T | 0.96(5.74e-02) | 0.87(2.68e-05) |
| Cord blood | CD4T | 0.88(6.69e-05) | 0.97(2.25e-01) |
| Cord blood | NK* | 0.73(1.24e-08) | 0.72(5.45e-09) |
| Cord blood | Bcell | 0.98(6.43e-01) | 0.95(2.29e-02) |
| Cord blood | Mono | 0.97(2.21e-01) | 0.93(3.43e-03) |
| Fetal placenta | Stromal | 0.97(8.87e-02) | 0.98(1.86e-01) |
| Fetal placenta | Hofbauer* | 0.76(2.31e-09) | 0.75(2.13e-09) |
| Fetal placenta | Endothelial | 0.96(2.57e-02) | 0.88(6.19e-06) |
| Fetal placenta | nRBC* | 0.81(6.28e-08) | 0.95(9.33e-03) |
| Fetal placenta | Syncytiotrophoblast | 0.98(1.82e-01) | 0.93(7.71e-04) |
| Maternal placenta | Trophoblasts | 0.98(2.72e-01) | 0.89(2.60e-05) |
| Maternal placenta | Stromal | 0.97(1.62e-01) | 0.97(1.72e-01) |
| Maternal placenta | Hofbauer* | 0.87(3.28e-06) | 0.74(1.05e-09) |
| Maternal placenta | Endothelial | 0.95(5.96e-03) | 0.98(2.56e-01) |
| Maternal placenta | nRBC | 0.92(1.96e-04) | 0.96(2.48e-02) |
| Maternal placenta | Syncytiotrophoblast | 0.98(4.04e-01) | 0.95(4.55e-03) |
| Fetal placenta | Trophoblasts | 0.99(6.88e-01) | 0.61(3.29e-12) |
| Values represent Shapiro-wilk test statistic and corresponding p-value in the parenthesis. *-did not reach statistical significance for normality either on natural or log_10_ scale. | | | |


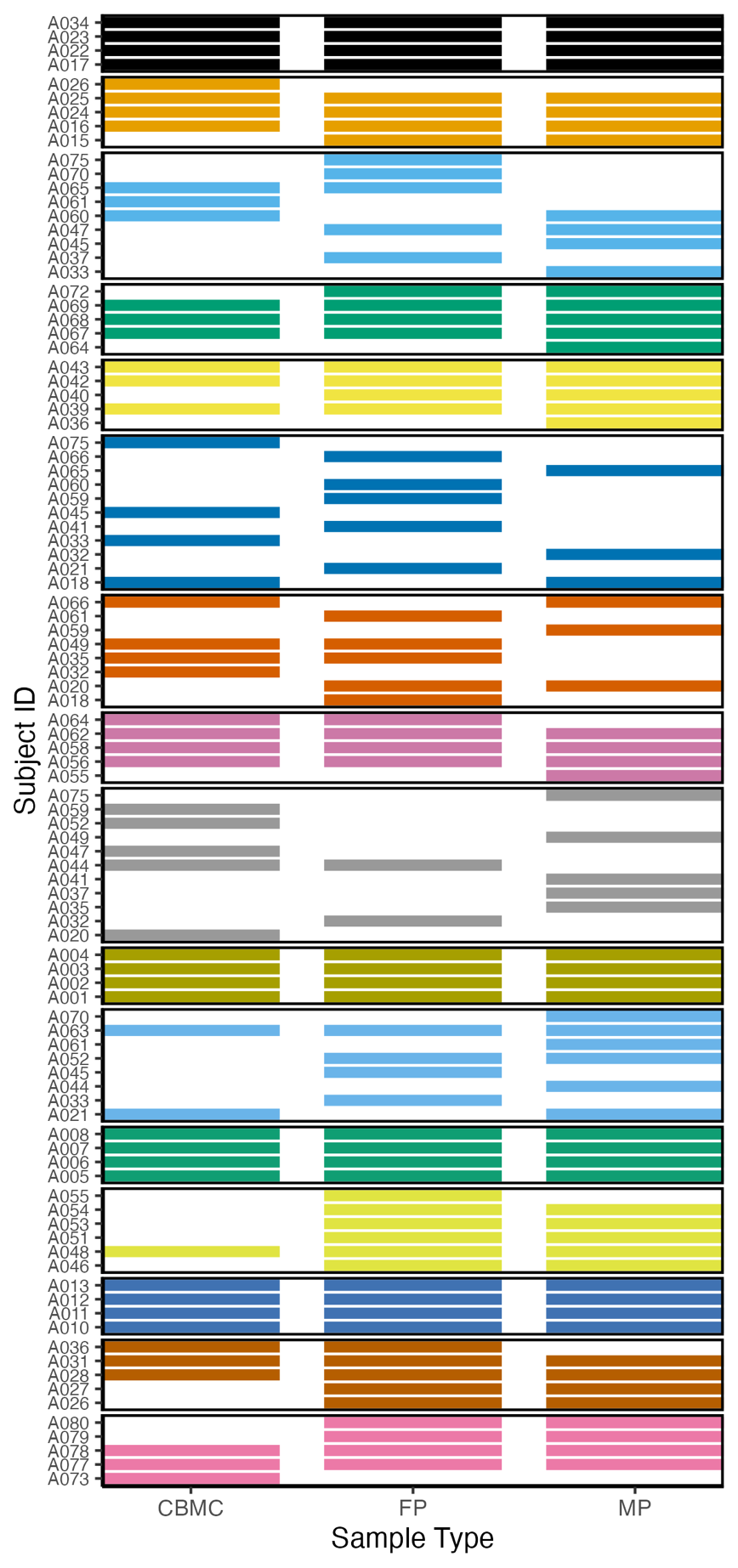
Figure S1. Arrangement of DNA samples to HM450 chips (n=16). The figure panel border and distinct colors represent HM450 chips. The sequence within a chip panel border does not represent the well positions. The y-axis represents the subject identifier.


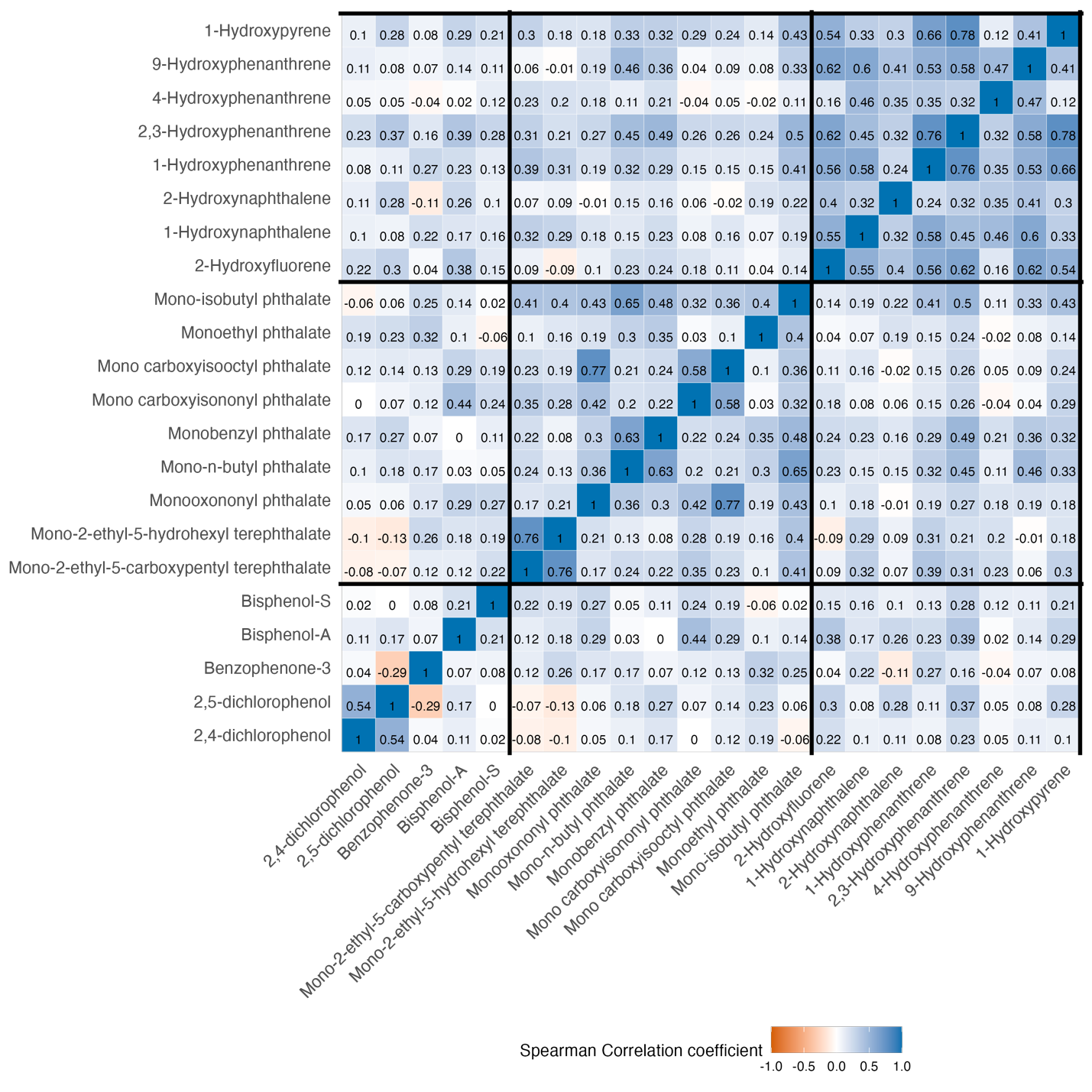
Figure S2: Spearman correlation coefficients between log_10_ transformed concentrations of maternal urinary biomarkers.


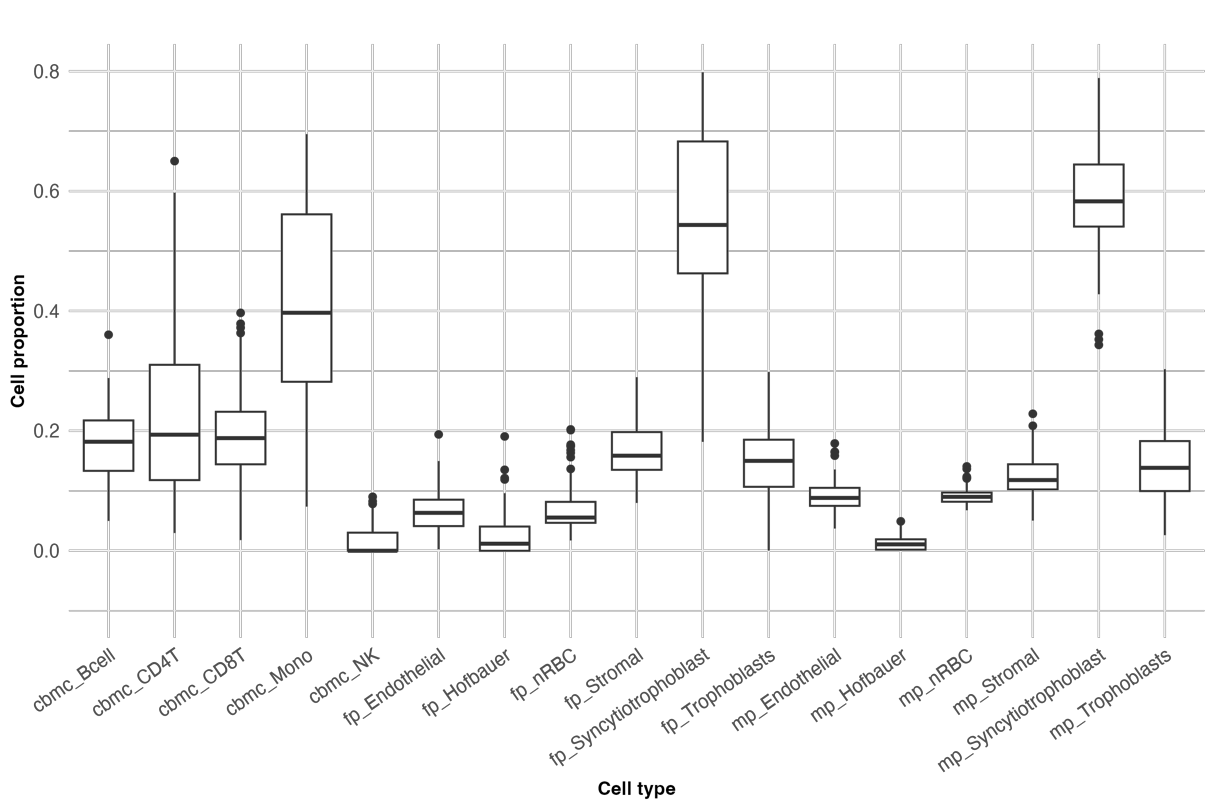


Figure S3: Distribution of cell proportions estimated using methylation intensities from CBMC and placenta (maternal and fetal sides). This plot showcases the median and interquartile range.


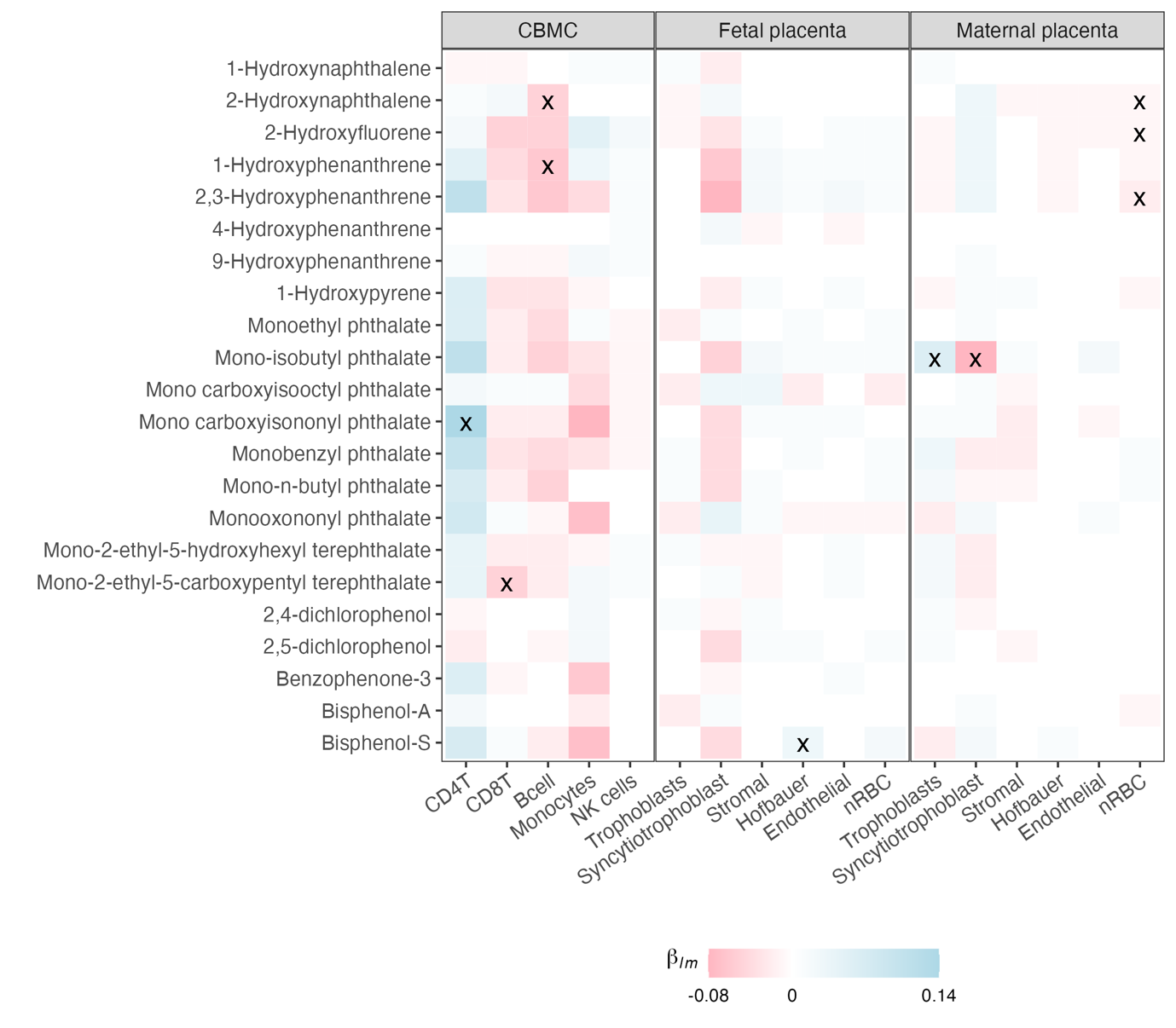
Figure S4-A. Association between maternal biomarker concentrations and estimated cell composition. The y-axis contains biomarkers measured from maternal urine at the delivery visit, and the x-axis contains cell proportions estimated using DNA methylation data. The color legend represents the beta coefficients calculated using linear regression. Beta coefficients in the red palette represent negative coefficients, and in blue represent positive coefficients. The regression estimates with 95% confidence intervals that do not contain null values are represented using “x”.


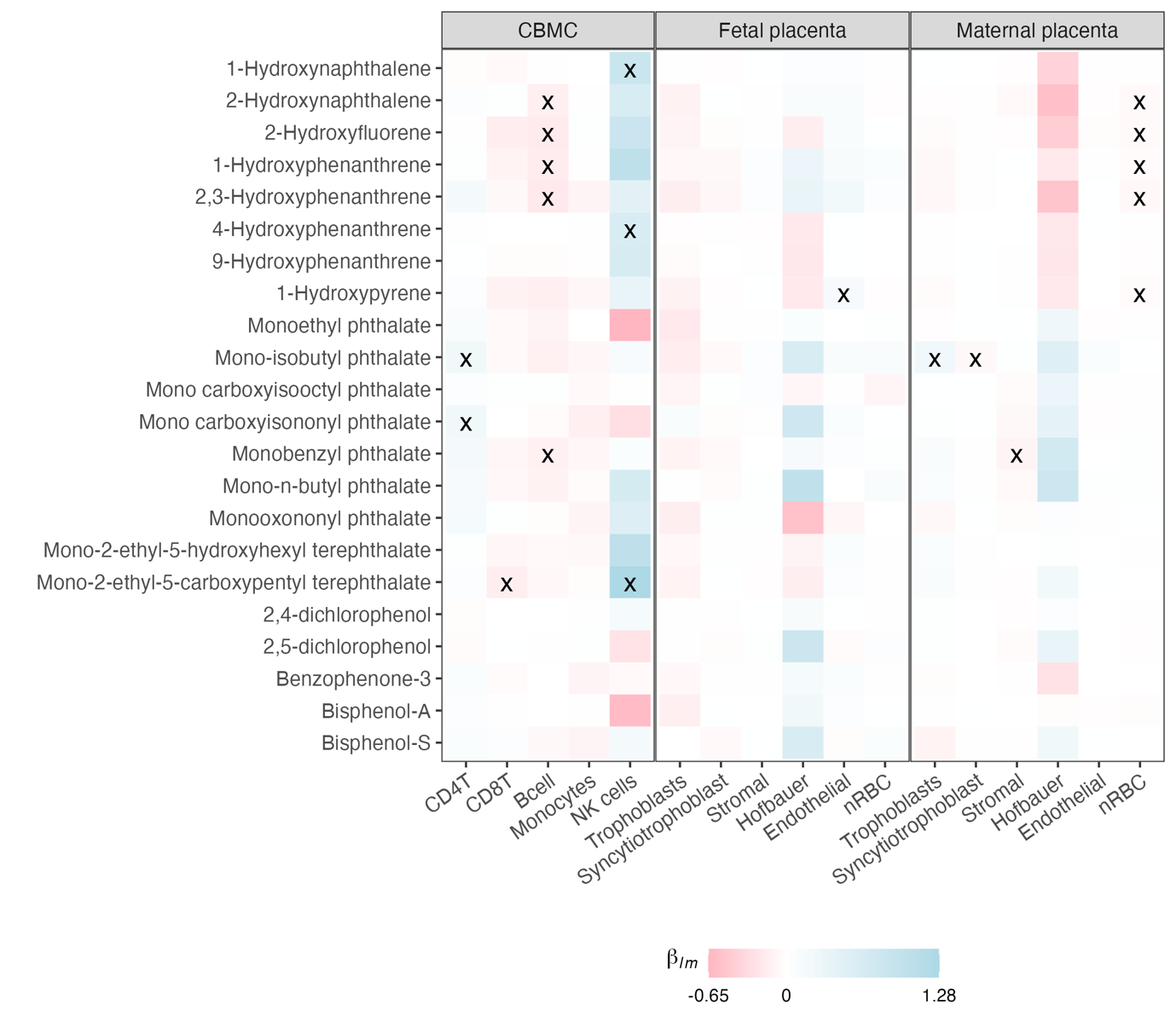
Figure S4-B. Sensitivity analysis – Association between maternal urinary biomarkers and log_10_ transformed cell proportions estimated from methylation intensities. In this figure, we presented the linear regression results by transforming the maternal urinary concentrations and cell proportions to the log_10_ scale.

Supplement methods (SM1): Extraction of placenta tissue: We collected the placenta tissue biopsies by placing the placenta on an absorbent pad with the fetal side (or cord insertion) up, as illustrated in Figure SM. 1-A. We then identified two locations on the tissue (one on the left [a] and the other on the right [b] side) with fewer or no veins to obtain 2 core placenta samples (A and B) about 2 cm (width), 2 cm (height), and 3 cm deep. We then vertically dissected the placenta tissue into equal-sized samples, with each side about 1-1.5 cm deep. The tissue with amnion and chorion was considered as the fetal side of the placenta tissue. We then placed the tissue samples on gauze to adsorb as much of the blood as possible and quickly transferred the placenta tissue samples into 2 ml sterile cryovials. The tissue samples for DNA methylation were stored in a -70° C freezer. We then used AFD (left-side fetal DNA sample) and AMD (left-side maternal side DNA sample) or BFD (right-side fetal DNA sample) and BMD (right-side maternal DNA sample) placenta tissue samples to perform DNA methylation experiments.

SM.1-A. Placenta tissue biopsy for DNA methylation (Adapted from a protocol from the University of Iowa)
